# What Has Genomics Taught An Evolutionary Biologist?

**DOI:** 10.1016/j.gpb.2023.01.005

**Published:** 2023-01-28

**Authors:** Jianzhi Zhang

**Affiliations:** Department of Ecology and Evolutionary Biology, University of Michigan, Ann Arbor, MI 48109, USA

**Keywords:** Variation, Interaction, Selection, Evolution, Genetics, Mutation

## Abstract

Genomics, an interdisciplinary field of biology on the structure, function, and **evolution** of genomes, has revolutionized many subdisciplines of life sciences, including my field of evolutionary biology, by supplying huge data, bringing high-throughput technologies, and offering a new approach to biology. In this review, I describe what I have learned from genomics and highlight the fundamental knowledge and mechanistic insights gained. I focus on three broad topics that are central to evolutionary biology and beyond—**variation**, **interaction**, and **selection**—and use primarily my own research and study subjects as examples. In the next decade or two, I expect that the most important contributions of genomics to evolutionary biology will be to provide genome sequences of nearly all known species on Earth, facilitate high-throughput phenotyping of natural variants and systematically constructed mutants for mapping genotype–phenotype–fitness landscapes, and assist the determination of causality in evolutionary processes using experimental evolution.

## Introduction

I was completing my first year as a doctoral student in genetics at Pennsylvania State University when the genome sequence of the pathogenic bacterium *Haemophilus influenzae*—the first from any free-living organism—was published in the summer of 1995 [1]. I remember to this day the circular genome illustrated on the cover of *Science* and the excitement that genome sequencing brought to us in the laboratory of Dr. Masatoshi Nei. At the time, I was studying the evolution of animal homeotic genes [2] that had been sequenced from diverse species through laborious cloning. It was unimaginable then that, in less than two decades, researchers would sequence the entire animal genome to acquire the sequences of specific genes such as homeotic genes for evolutionary studies [3]. This drastic change in the approach to gene sequence acquisition in the study of evolution is a testament of the enormous advance and broad impact of genomics.

Although initially concentrating on genome sequencing, genomics has expanded substantially in its scope. Hereinafter, I use genomics to refer to an interdisciplinary field of biology on the structure, function, and evolution of genomes. Hence, it encompasses many subjects, including, for example, genome sequencing and annotation, transcriptomics, proteomics, and metabolomics. In my view, genomics has revolutionized life sciences, including my field of evolutionary biology, by supplying huge data, bringing high-throughput technologies, and offering a new approach to biology. For example, before the genomic era, phylogenetic relationships among species were usually inferred based on one to a few genes [4]; nowadays, phylogenies are typically inferred from dozens to hundreds of genes, often encompassing all orthologous genes from the relevant genomes or transcriptomes [5], [6], [7], [8]. High-throughput technologies developed by genome biologists such as ribosome profiling and proteomics have helped answer long-standing evolutionary questions such as the nature of the selections influencing synonymous codon usage [9], [10], [11], [12]. Speciation and species divergence are now routinely studied by comparing genomes of related species, revealing previously underappreciated processes such as introgression [13].

As someone who has witnessed the continued impact of genomics on evolutionary biology and whose research has benefited greatly from genomics, here I review the most important things that I have learned from nearly three decades of discoveries in genomics (Table 1). Evolutionary biology is a gigantic field, so my experience and views by no means represent those of all evolutionary biologists. For example, the interaction between cancer genomics and evolutionary biology has created the cancer evolution field that aims to understand both cancer and the evolution of cells within an organism [14], but I will not discuss it here. I hope that my review will help the further infusion of genomics into evolutionary biology and stimulate evolutionary thinking in genomic research.

**Table 1:** **Main topics discussed**

**General topic**		**Specific topic**
Variation	Genome size and gene structure	Variation in genome size; variation in intron density and size; causes of these variations
	RNA and protein production	Alternative transcriptional initiation; alternative splicing including back-splicing; alternative polyadenylation; RNA editing; alternative translation initiation; stop codon readthrough; causes of these variations
	Protein evolutionary rate	Determinants of the protein evolutionary rate; E–R anticorrelation and its multiple causes; meaning of functional constraint
Interaction	Protein interaction	Evolutionary rate of protein interaction with and without gene duplication; features of protein interaction networks (essential nodes and edges, modularity); importance of protein complexes
	Genetic interaction	Intragenic epistasis; intergenic epistasis; pairwise *vs.* high-order epistasis; idiosyncratic epistasis and consequences; offspring fitness as a function of mating distance
	G × E interaction	G × E interaction and environmental pleiotropy; antagonistic environmental pleiotropy; prevalence of G × E; adaptation in a changing environment
Selection	Convergence	Testing molecular convergence; phenotypic *vs.* molecular convergence; echolocation and prestin
	Gene expression noise	Deleterious expression noise; beneficial expression noise; fitness noise; intrinsic *vs.* extrinsic expression noise; expression noise and dose imbalance; mechanisms alleviating the detriment of noise
	Mutation rate and spectrum	Selections acting on the genomic mutation rate; mutation spectrum seems to be subject to selection; no optimization of gene-specific mutation rates

*Note*: E–R anticorrelation, the strong negative correlation between the expression level of a gene and its protein evolutionary rate; G × E interaction, gene-by-environment interaction.

Realtors often tell customers that the three most important things to consider when buying a house are “location, location, location”. I will imitate this phrase to emphasize the themes of the lessons I learned from genomics.

## Variation, variation, variation

If general laws such as Newton’s three laws of motion characterize physics, variation is probably the most prominent feature of the living world. Biology has laws, but no law seems to be general. Take for example the three Mendelian laws of inheritance: dominance, segregation, and independent assortment. Complete dominance of one allele to another as in the inheritance of round *vs.* wrinkled peas in Mendel’s experiments is uncommon (see [15] for the molecular genetic basis of the wrinkled pea phenotype). In most cases, dominance of one allele to another is incomplete. Equal segregation of the two alleles is relatively general, but exceptions are known in the form of segregation distortion in which the two alleles at a locus are not inherited with the same probability [16]. Independent assortment applies only to genes located on different chromosomes, so it is frequently violated. Genomics has uncovered both degrees and types of variations that were unknown to biologists and has helped decipher the general principles governing the patterns of some of these variations. To me, the most interesting examples are as follows.

### Genome size and gene structure

Although eukaryotes tend to have larger genomes than prokaryotes, exceptions abound because of the huge variation across prokaryotes as well as across eukaryotes. For instance, *Nasuia deltocephalinicola*, an endosymbiotic bacterium of insects, has a genome of only 112 kb [17], whereas the soil-dwelling bacterium *Sorangium cellulosum* has a genome of 14.8 Mb [18]. The lower end of the eukaryotic genome size variation is believed to be the microsporidian *Encephalitozoon intestinalis*, an obligate intracellular opportunistic fungus with a genome of 2.3 Mb, whereas the higher end includes the flowering plant *Paris japonica* (149 Gb), fern *Tmesipteris obliqua* (147 Gb), and lungfish *Protopterus aethiopicus* (130 Gb) [19]. The tiny genomes of endosymbionts are generally thought to be consequences of losses of genes unnecessary for the endosymbiotic life that is in a large part dependent on the host [20]. The huge genomes of the eukaryotes mentioned are filled with transposons, probably due to unusually high transposon activities and/or a lack of sufficient selection suppressing/removing the transposons [21]. Hence, compared with genomes of intermediate sizes, the extremely small genomes have reduced functions (evident from gene losses), but the extremely large genomes do not seem to show increased functions.

In eukaryotes, the genome size variation is often accompanied by gene structure variations, most notably in intron density and size. Although known to some extent before eukaryotic genomes were sequenced, the magnitude of this variation revealed by genome sequencing is astounding. For example, over 90% of human genes have introns, but only about 5% of budding yeast (*Saccharomyces cerevisiae*) genes are intron-containing. Although the average length per intron (6.9 kb) is 43 times that per exon (160 bp) in humans [22], they are both around 450 bp in the fruit fly *Drosophila melanogaster* [23]. Such variations across species have been explained by a variation in effective population size, which determines the intensity of purifying selection against slightly deleterious mutations, and a variation in mutation bias [21].

### RNA and protein production

The basic process of RNA and protein production through transcription and translation was worked out by molecular biologists long before the genomic era. However, genomics has unveiled tremendous variations at virtually every step of RNA/protein production. For example, unlike what we learned from textbooks years ago that each gene has one transcription start site, we now know that an average human gene has four transcription start sites [24], such that a pool of transcripts with heterogenous 5′ ends are often synthesized from each gene. In addition to the widely known alternative splicing that can create multiple mRNA isoforms from a transcript, alternative polyadenylation is also common—about 50% of human genes have at least three polyadenylation sites per gene [25]. Besides linear splicing, RNAs may also be back-spliced to create circular RNAs, which are usually more stable than linear RNAs; over 50% of human protein-coding genes are known to produce circular RNAs [26]. RNA editing, which enzymatically alters the RNA sequence (excluding RNA processing such as splicing, 5′-capping, and 3′-polyadenylation), has been known since the 1980s [27]. However, it is genome and transcriptome sequencing that has revealed both the diversity and prevalence of RNA editing [28], [29], [30]. For instance, adenosine (A)-to-inosine (I) editing occurs at about two thousand coding sites [31] and millions of non-coding sites in the human genome [32], and this is but one of over 160 different types of RNA editing documented thus far [29]. In protein synthesis, we now know that translational initiation could occur at one of several positions that need not be occupied by ATG [33]. For example, on average 2.5 translation initiation sites per gene have been observed in just one human cell line [34]. Even at the supposed end of protein synthesis, ribosomes may occasionally bypass the stop codon, creating an extended peptide. This phenomenon of stop codon readthrough has been observed for over 300 fruit fly genes [35].

These variations generate a huge diversity in the RNA and protein products of a single gene, far beyond the notion that one gene encodes one protein. These variations are both exciting and puzzling. They are exciting because they could potentially explain why complex organisms such as mammals need only about 20,000 (annotated) protein-coding genes. They are puzzling because many of the variations are not evolutionarily conserved, suggesting the possibility that they are functionally unimportant, a notion that is supported by a number of features of these variations [36]. For example, these variations tend to be greater in more weakly expressed genes [36], resembling features of mistranscription [37] (*i.e.*, incorporation of wrong nucleotides in transcription) and mistranslation [11] (*i.e.*, incorporation of wrong amino acids in translation). There is generally no evidence that the lack of evolutionary conservation of these variations reflects lineage-specific adaptations. The emerging consensus is that most of these variations likely reflect molecular errors in transcription, RNA processing, and translation, much like mistranscription and mistranslation, whereas only a minority may be adaptive [36]. Here, evolutionary thinking and analysis can help differentiate functional from nonfunctional variations in RNA/protein production that are so abundantly unraveled by powerful genomic technologies [36]. The widespread presence of molecular errors in cellular processes as fundamental as transcription and translation attests the imperfection of cellular life and the limitation of natural selection.

### Protein evolutionary rate

It has been known from the 1960s that different proteins encoded in the same genome can have drastically different rates of sequence evolution; this variation has been explained primarily by a variation in functional constraint across proteins [38]. Exactly what factors determine the functional constraint, however, has been elusive. At the turn of the century, protein evolutionary rates were computed for large numbers of genes. Surprisingly, despite the large data size, the evolutionary rate of a protein was found to be barely correlated with its functional importance assessed by the phenotypic or fitness effects of gene deletion [39], [40]. Instead, in all three domains of life, the mRNA level of a gene seems to be the major determinant of the rate of protein sequence evolution, although many minor determinants exist [41], [42]. A series of hypotheses have been proposed to explain the strong negative correlation between the expression level of a gene and its protein evolutionary rate (*i.e.*, the E–R anticorrelation) [42]. For example, the protein misfolding avoidance hypothesis posits that the anticorrelation results from selection against protein misfolding. Specifically, under the assumption that proteins occasionally misfold and that misfolded proteins are cytotoxic, one can infer that selection for a lower protein misfolding probability is stronger on highly expressed genes than on lowly expressed ones, because the same misfolding probability corresponds to more misfolded molecules for highly expressed than lowly expressed genes [43], [44]. Another hypothesis posits that the selection arises from protein misinteraction avoidance [45]. More recently, we found that coding mutations in highly expressed genes are more likely to reduce the mRNA level of the gene than those in lowly expressed genes, and because reducing the mRNA level of a gene is often deleterious, highly expressed genes are selectively constrained relative to lowly expressed ones [46]. Empirical evidence for each of these hypotheses exists, suggesting that they all contribute to the E–R anticorrelation. This said, it is unclear which of the proposed mechanisms makes the greatest contribution to the E–R anticorrelation and whether all causes of the anticorrelation have been accounted for. Regardless, this line of research has greatly improved our understanding of “functional constraint” in protein evolution. Apparently, the word “function” in “functional constraint” includes not only physiological function but also toxicity or negative function. This model of protein evolution considerably broadens the standard model that has been around for approximately a half century. These findings on the causes of the huge variation in evolutionary rate among proteins would not have been possible without genome sequences, genome-scale gene expression measures, and various functional genomic data.

## Interaction, interaction, interaction

Although variation is likely the most prominent feature of the living world, interactions among various components of a biological system undoubtedly play a critical role in the functioning of the system. Yet, interactions among genes or proteins, necessary for the functioning of cells and organisms, were rarely studied in a systematic fashion prior to the genomic era. Genomics brought tools and resources necessary for investigating such interactions at a grand scale and was largely responsible for the birth of systems biology, which in my view is a study of interactions.

Protein interactions refer to physical interactions between proteins, whereas genetic interactions (also known as epistasis) refer to the phenomenon that the phenotypic effect of a mutation depends on the presence or absence of another mutation. Genome-scale protein interaction data provide unprecedented opportunities for systematically studying protein function evolution. Large data of epistasis allow testing many evolutionary models that involve epistasis. For example, the Dobzhansky–Muller model of reproductive isolation and speciation requires negative epistasis between two genes one from each of the two species concerned [47], and the mutational deterministic hypothesis of the evolution of sexual reproduction relies on negative epistasis between deleterious mutations [48]. In addition, genomics has stimulated the study of gene-by-environment (G × E) interactions, which play multiple important roles in evolutionary biology. Below I discuss patterns of protein interactions, genetic interactions, and G × E interactions revealed by genomics and their evolutionary implications.

### Protein interactions

Although the evolutionary rate of a protein can be easily measured at the level of protein sequence and then compared among different proteins, it is difficult to do the same at the level of protein function, primarily because different proteins have different functions that are not easily comparable quantitatively. Nonetheless, most proteins interact with at least one other protein, so one could determine protein interactions in a high-throughput fashion and compare these interactions among proteins. This is, however, easier said than done, because there are many experimental systems for determining protein interactions and because different systems (or different laboratories using the same system) often yield different results [49], [50]. A functional genomics laboratory is typically interested in measuring protein interactions in only one species, so it is difficult to compare data from different species that are usually generated by different laboratories that often use different experimental systems. More than a decade ago, my group used a low-throughput approach to measure protein interactions for a set of orthologous genes from two yeast species in order to estimate the evolutionary rate of protein interactions [51]. We found that protein interactions are extremely conserved, with an evolutionary rate of 2.6 × 10^−10^ per protein interaction per year, three orders of magnitude lower than the rate of protein sequence evolution measured by the number of amino acid substitutions per protein per year. That is, most amino acid substitutions do not alter protein interaction partners.

Protein interactions presumably evolve more rapidly after gene duplication than without duplication, because paralogous genes present in a genome and created by past gene duplication events often exhibit somewhat different functions including protein interactions [52], [53]. Although we initially planned to compare the evolutionary rate of protein interactions in the presence and absence of gene duplication, the aforementioned low-throughput experiment was exhausting, forcing us to abandon the plan. To my knowledge, such a comparison has not been done to this day. Instead, we compared duplicate genes of different evolutionary ages identified from the same genome, an analysis that required protein interaction data from only one species [53]. Interestingly, the shared number of protein interactions between a pair of paralogs typically drops quickly with evolutionary time since duplication, whereas the total number of distinct interactions for the gene pair gradually rises and eventually approaches that for two singleton genes. These temporal patterns suggest rapid subfunctionalization—partition of ancestral functions—after gene duplication, as well as gradual neofunctionalization—gain of new function. Notably, the amount of neofunctionalization is substantial, because a pair of paralogs start with the number of protein interactions of one gene but eventually possess almost the total number of interactions of two genes. Notwithstanding, there are old paralogous genes that still share functions and interaction partners, a phenomenon that has been explained by expression reductions [54] and structural/functional entanglements [55] that hinder the functional divergence between paralogs.

The accumulation of protein interaction data quickly led to the study of protein interaction networks in which each protein is represented by a node and two proteins are connected by an edge if they interact with each other. Important features of protein networks were characterized under the influence of the burgeoning network theory. For example, it was discovered that proteins with more interactions are more likely to be essential (*i.e.*, deleting the protein-coding gene is lethal) [56]. Although the essentiality of a protein could be caused by the joint effect of all of its interactions, my group proposed and provided evidence that a protein is essential because it has at least one essential interaction, expanding the concept of essential nodes to essential edges [57]. Another interesting network feature is modularity, meaning that the network can be divided into modules or communities and links within modules are much denser than those across modules. Protein networks are highly modular, but modules could arise as a byproduct of the network growth via gene duplication so do not necessarily represent functional units [58].

Members of a stable protein complex are usually considered to interact physically with one another either directly or indirectly. These interactions seem to be particularly important because many analyses have found them to be evolutionarily constrained and the interacting partners to coevolve. For example, whether a gene is essential or not is dependent on the genetic background (*i.e.*, other genes in the genome), and the essentiality of a gene can vary among different strains of a species. Interestingly, this variation is often coordinated among members of the same protein complex [59]. Another example is that genes encoding members of the same protein complex tend to be correlated in their duplication history (*i.e.*, they either all duplicate or all resist duplication), presumably reflecting a requirement for dose balance among protein complex members [60], [61]. Similarly, such dose balance is manifested in the dosage compensation of genes encoding members of stable protein complexes (but rarely other genes) in the origin of the X chromosome of placental and marsupial mammals [62]. In the same vein, genes encoding components of the same protein complex tend to have reduced intrinsic expression noise [63] and be chromosomally linked, likely resulting from natural selection for intracellular among-component dose balance [64] (see below).

### Genetic interactions

There are many examples of genetic interaction or epistasis in the classic Mendelian genetics literature in which one mutation suppresses or enhances the phenotypic effect of another mutation. Metabolic pathways and networks known from biochemistry also suggest the prevalence of epistasis. That many proteins interact physically and many residues within a protein interact physically further suggests the abundance of epistasis. Formally, epistasis is usually measured by ε = *f*_AB_ − *f*_A_ × *f*_B_, where *f*_A_ and *f*_B_ are the phenotypic values of a trait relative to that of the wild type for mutants A and B, respectively, and *f*_AB_ is the phenotypic value of the corresponding double mutant. Although the trait of concern can vary, evolutionary biologists are most interested in fitness or proxies of fitness. Epistasis is said to be positive when ε > 0 and negative when ε < 0. If fitness is the trait of concern, positive epistasis means that the double mutant is fitter than expected from the two constituent mutations combined under no epistasis, whereas negative epistasis means that the double mutant is less fit than the expectation. For convenience, I will separately discuss epistasis within genes (intragenic epistasis) and that between genes (intergenic epistasis), because the methods for probing them are often different.

Intragenic epistasis is typically probed by creating many mutants of a gene, each containing one to a few mutations, followed by phenotyping of these mutants. Next-generation sequencing of barcodes associated with different mutants (bar-seq) offers an efficient way to measure the frequencies of many genotypes in a population. Bar-seq of the population before and after the competition among genotypes (mutants and the wild type) provides estimates of mutant fitness relative to the wild type, which could then be used to estimate epistasis. The gene or gene segment of interest can even serve as the barcode if it is short enough to be covered by a sequencing read. For example, using this approach, my lab measured the fitness of over 65,000 mutants of a yeast tRNA gene (72 nt) under a stressful laboratory condition [65]. We measured epistasis for 12,985 pairs of mutations and found 42% of them to be statistically significant. Interestingly, epistasis in the tRNA gene is negatively biased, with 86% of the significant epistasis values negative. With negative epistasis, each mutation tends to be more detrimental (or less beneficial) in the presence than absence of another mutation. Furthermore, because most mutations are deleterious, accumulation of a few random mutations in the wild type could drastically lower the fitness. Although a negative bias in intragenic epistasis has also been observed from another RNA gene [66] and several protein-coding genes [67], [68], [69], more data are needed to confirm that this is general.

Thus far, systematic surveys of intergenic epistasis have almost exclusively used null mutations. In other words, our systematic knowledge about intergenic epistasis largely comes from that between a null mutation of one gene and a null mutation of another gene. For example, such intergenic epistasis has been mapped for 23 million (of a total of 36 million) gene pairs in budding yeast [70]. What does intergenic epistasis tell us about the functional relationship between genes? If two genes are functionally redundant or overlapping (*e.g.*, encoding enzymes in alternative pathways for synthesizing the same product), deleting either gene would have a much smaller functional effect than deleting both genes, creating negative epistasis. Hence, negative epistasis between two genes suggests their functional redundancy. By contrast, if two genes are interdependent in performing a function (*e.g.*, encoding two indispensable components of a protein complex, or two enzymes in a linear metabolic pathway), deleting either gene would have a similar functional effect as deleting both, creating positive epistasis. Hence, positive epistasis between two genes suggests that they make distinct, interdependent contributions to a function. Additionally, epistasis between any gene and an essential gene is positive, regardless of their functional relationship, because deleting both genes does not lower fitness more than deleting the essential gene. The simple logic in the scenarios above has been modeled using flux balance analysis of metabolism to allow quantitative predictions of epistasis between metabolic genes, which have subsequently been validated experimentally [71]. Because intergenic epistasis is commonly assessed by deleting the two genes of interest individually and jointly, it is unclear whether a different level or even different sign of intergenic epistasis will occur between non-null mutations. If the sign of intergenic epistasis depends on the specific mutations examined, one wonders what the sign of intergenic epistasis between non-null mutations tells us about the functional relationship of the two genes (or mutations). The genomic technology is now available for addressing this fundamental question.

Although our discussion has focused on epistasis between two mutations, epistasis can also occur among three or more mutations (*i.e.*, high-order epistasis). Empirical data of high-order epistasis (*e.g.*, see [55]) are scarce for two reasons. First, the space for *n*^th^-order epistasis among *M* mutations (*i.e.*, number of *n*-mutation combinations) is $\left(\frac{M}{n}\right)$, which is huge for large *M* and *n*; hence high-order epistasis is difficult to map systematically. Second, one must phenotype 2*^n^* genotypes in order to estimate *n*^th^-order epistasis [72]. For example, 2^3^ = 8 genotypes (three single mutants, three double mutants, one triple mutant, and the wild type) must be phenotyped to estimate the 3rd-order epistasis among three mutations. As *n* increases, both the labor for phenotyping 2*^n^* genotypes and the estimation error of the *n*^th^-order epistasis become unbearably high.

Analysis of several (intragenic and intergenic) phenotypic or fitness landscapes found that epistasis is highly idiosyncratic, meaning that the same mutation can have drastically different phenotypic/fitness effects when occurring in different genotypes [72]. This idiosyncrasy is responsible for a number of evolutionary phenomena that sometimes look contradictory to one another [72]. For example, under idiosyncratic epistasis, a beneficial mutation tends to have a smaller benefit when occurring in a fitter genotype than in a less fit genotype, creating the so-called diminishing returns epistasis, which is negative epistasis, in adaptations. Idiosyncratic epistasis also makes a deleterious mutation on average less deleterious when occurring in a less fit genotype than in a fitter genotype, creating positive epistasis between deleterious mutations in mutation accumulation experiments. Theory shows that the aforementioned different impressions from experimental evolution (*i.e.*, adaptation) and mutation accumulation about the pattern of epistasis do not necessarily tell us about the shape of the fitness landscape or the relative abundance of positive *vs.* negative epistasis but can both be consequences of the idiosyncrasy of epistasis [72].

An important implication of negative epistasis between natural genetic variants is that it could lead to genetic incompatibility, causing reduced fitness of the hybrid compared with homozygous parents. The presence of such negative epistasis within a species can be assessed by studying the hybrid fitness as a function of the mating distance—sequence divergence between its parental genomes. We observed from plant, animal, and fungal model organisms that the hybrid fitness is an inverted U-shaped function of the mating distance and peaks when the mating distance is slightly greater than the mean divergence between conspecifics [73]. This finding confirms the existence of intraspecific genetic incompatibility and shows that the benefit of heterosis (*i.e.*, hybrid vigor) is at least partially offset by the harm of genetic incompatibility even within species.

### G × E interactions

G **×** E interactions refer to the phenomenon that a mutation has different phenotypic effects under different environments. The fact that no organism outcompetes all other organisms in all environments is presumably due to G **×** E interactions, which lead to environment-specific fitness ranks among genotypes. G × E interactions are related to the concept of pleiotropy, which is the phenomenon that one mutation influences multiple traits [74]. Because a trait such as the growth rate depends on the environment, it is conventional at least among those studying microbes to treat a trait in multiple environments as multiple traits. Hence, if a mutation influences the growth rate in more than one environment, the mutation is said to show (environmental) pleiotropy, and if the mutational effect varies among environments, the mutation is said to exhibit G **×** E interaction. If the effects of a mutation in two environments are in opposite directions, the mutation is said to show antagonistic pleiotropy, which is also G **×** E interaction. Genomic tools have allowed creating large numbers of mutants and phenotyping them in multiple environments, which have considerably improved our understanding of the prevalence and evolutionary consequences of G **×** E interactions and environmental pleiotropy.

Although deleting a gene typically lowers the organismal fitness, my lab found in a screen of all nonessential yeast genes in six different environments that, in each environment, several hundred genes increase the fitness when deleted [75]. The fact that these genes exist in the yeast genome suggests that deleting them may be detrimental in other environments. Indeed, deleting them tends to lower the fitness in one or more of the other environments examined [75]. If the presence of a gene is detrimental in an environment, natural selection should suppress its expression in the environment if appropriate regulatory mutations are available. As predicted, we found evidence for such regulatory evolution in strains that have been in the environment of interest for a sufficiently long time [75]; nevertheless, the gene is still intact in these strains, probably because these strains additionally experience environments in which the gene function is beneficial.

Studies of fitness effects of point mutations in multiple environments also revealed the prevalence of G × E interactions. For example, depending on the pair of environments compared, 18%–66% of point mutations in a yeast tRNA gene show significant G × E interactions [76]. From the fitness effects of thousands of nonsynonymous mutations in 21 yeast genes under four different environments, it was found that among those mutations that are significantly beneficial in at least one environment, 70.3% are significantly deleterious in at least one other environment [46].

G × E interactions are also abundantly observed among natural polymorphisms. For example, using 1005 segregates generated from a cross between two yeast strains, we mapped quantitative trait loci (QTLs) for growth rates in 47 different environments. On average, 58% of QTLs identified in two environments exhibit G × E interactions between the two environments [77]. Most G × E interactions show concordant effects between environments, but, as the effect size of a QTL in one environment enlarges, the probability of antagonism in the other environment increases [77]. In a more sophisticated study, we mapped QTLs that simultaneously impact two important parameters of yeast population growth: maximum growth rate *r* (growth rate when the population is very small) and carrying capacity *K* (maximum population size that can be sustained in the environment) [78]. We found that, depending on the environment, a QTL may concordantly or antagonistically impact *r* and *K*. Furthermore, the antagonism becomes more common as the quality of the environment measured by the average *r* of all genotypes rises. Consequently, *r* and *K* tend to show tradeoffs in relatively rich environments but tradeups in relatively poor environments. These contrasting trends are probably generated by the relative impacts of two factors—the tradeoff between the speed and efficiency of ATP production and the energetic cost of cell maintenance relative to reproduction [78].

An important implication of G × E interactions is that, because the natural environment changes frequently, the fitness effect of a mutation may vary greatly in its lifetime (*i.e.*, from its appearance in a population to its fixation or loss). Under antagonistic pleiotropy, beneficial mutations in one environment contribute to the adaptation to that environment but may never get fixed if they soon become deleterious in the next environment. Consequently, antagonistic pleiotropy can conceal adaptations in changing environments. My laboratory performed experimental evolution of yeast in changing environments as well as corresponding constant environments and found supporting evidence for the hypothesis above [79].

## Selection, selection, selection

Ever since Darwin, selection has been a central topic of evolutionary biology. Before we go on, however, it is important to clarify two types of selection: positive and negative. Positive selection promotes the spread and fixation of beneficial mutations, whereas negative (or purifying) selection prevents the spread and fixation of deleterious mutations [80]. In the field of molecular evolution, the advent of the neutral theory [38] in the late 1960s and the neutralist–selectionist debate that followed made neutrality-testing and (positive) selection-detection popular subjects of investigation. Prior to the genomic era, statistical evidence for positive selection acting on one gene was often sufficient for a publication. The availability of genome sequences from related species stimulated genome-wide searches of positive selection signals (*e.g.*, [81]). Although the fraction of genes in a genome found to have been positively selected is usually small, the absolute number of positively selected genes reported per genome is often quite large. Although the state of the neutral theory as an accurate description of gene and genome evolution is debated [82], [83] and probably will remain controversial for the foreseeable future, below I briefly discuss three selection-related subjects that have seen substantial developments thanks to genomics.

### Convergence

Convergence refers to the phenomenon that a particular state originates in more than one evolutionary lineage. Convergence may be classified into convergent evolution and parallel evolution; they differ in that the ancestral states in different lineages are the same in parallel evolution but different in convergent evolution [84]. Evolutionary biologists are interested in convergence because the probability of origination of a complex trait multiple times without a common selection is presumably extremely low; hence, convergence suggests a common selection in multiple lineages [85]. Furthermore, convergence suggests a limited number of solutions to a problem [85]. At the protein sequence level, however, convergence may not be too rare even without common selections because each position in the sequence has only 20 choices. For this reason, a statistical test of protein sequence convergence that computes the chance probability of the observed convergence was developed [84]. This test was later revised by considering the amino acid composition variation across sites, because this variation increases the false-positive rate if not taken into account [86]. Many proteins have now been reported to show levels of sequence convergence beyond the chance expectation. A striking example is the mammalian hearing protein prestin, which provides the electromobility of cochlear outer hair cells that is responsible for cochlear amplification, an active process that confers sensitivity and frequency selectivity to the mammalian auditory system. Sequence convergence in prestin is so strong that a phylogeny reconstructed using the prestin sequences clusters echolocating bats with echolocating whales in exclusion of nonecholocating bats [87], [88]. Subsequent experiments confirmed the functional importance of the parallel amino acid substitutions in prestin [89]. Such experimental demonstrations of the importance of convergent/parallel amino acid substitutions to the functional convergence of the proteins involved are, however, uncommon [90], and most studies of sequence convergence end after identifying sequence convergence.

Some authors have reported sequence convergence at the proteome scale between lineages that show certain morphological or physiological convergences [91], but because sequence convergence is possible by chance, there may not be causality between sequence and phenotype convergences. Indeed, the proteome-level sequence convergence is lower between echolocating bats and bottlenose dolphins (which echolocate) than that between echolocating bats and cows (which are relatively closely related to dolphins but do not echolocate) [92], [93]. Another complication is that the expected level of sequence convergence tends to decline with the divergence of the lineages compared, because due to epistasis the same amino acid at a position is less likely to have the same functional effect in more divergent lineages [86], [94]. Furthermore, discordance between gene trees and species trees caused by either incomplete lineage sorting or introgressive hybridization can cause mis-identification of sequence convergence [95]. Hence, although comparative genomics allows identifying sequence convergence at the proteome scale for many lineages [96], interpretations can be difficult.

### Gene expression noise

Gene expression noise refers to the variation in the expression level of a gene among isogenic cells in the same environment. I single out this trait for discussion because it is a variance trait whereas most phenotypic traits studied by biologists are mean traits such as the average body weight instead of the variance of the body weight of a species. Gene expression noise is caused by stochastic variations in molecular and cellular processes, although the magnitude of the noise is genetically determined. Gene expression noise, or more precisely protein concentration noise, has been measured at the genome scale in yeast [97] and *Escherichia coli* [98] thanks to genomics and high-through biology. To an evolutionary biologist, the foremost questions are whether expression noise is subject to natural selection and how expression noise evolves and affects evolution. These questions have been addressed in a series of studies. First, gene expression noise leads to imprecise cellular behaviors so is expected to be generally detrimental. For example, it may ruin the stoichiometric relationship among functionally related proteins such as members of a protein complex and may disrupt cellular homeostasis. Using flux balance analysis of metabolism, we predicted that expression noise reduces the mean fitness of a cell by at least 25% and that this reduction cannot be substantially alleviated by gene overexpression [99]. We also found that higher sensitivity of fitness to the expression fluctuations of essential genes than nonessential genes creates stronger selection against expression noise for essential genes [99], explaining why essential genes tend to be less noisy than nonessential ones [100]. The expression noise of a gene may be reduced by relocating the gene from a noisy to a quiet genomic region; indeed, essential genes are concentrated in genomic regions with inherently low expression noises (assessed using reporter genes) [101]. Second, theory predicts that elevated expression noise can be beneficial when the mean expression level is suboptimal and fitness is a convex function of the expression level [102]. Indeed, we found one and only one functional group of yeast proteins with unexpectedly high expression noise—plasma-membrane transporters; the high noise presumably reflects a bet-hedging strategy to deal with unpredictable environmental fluctuations. Third, gene expression noise generates fitness noise, which generally lowers the efficacy of natural selection similar to the effect of population shrinkage [99]. Fourth, gene expression noise can be separated into two components: extrinsic and intrinsic. The extrinsic noise arises from the among-cell variation in cell state such as the cell cycle stage or the concentrations of various transcription factors, whereas the intrinsic noise is due to the stochastic process of gene expression even under a given cell state such as the stochastic binding of a promoter to RNA polymerase. Using single-cell RNA sequencing data from hybrids of two mouse strains, we dissected the expression noise into extrinsic and intrinsic components for thousands of genes [63]. Gene function-associated noise trends suggest different selections on intrinsic and extrinsic noises. For instance, because dose balance is important for protein complex members as mentioned earlier and because extrinsic noise does not create dose imbalance as long as members of the same protein complex are co-regulated in expression, protein complex members should have reduced intrinsic noise but not necessarily reduced extrinsic noise. These predictions have been empirically validated [63]. Genes controlling the cell cycle should express differently at different cell cycle stages. However, within a cell that is at a particular cellular stage, cell cycle genes should preferably show consistent expression. Indeed, compared with other genes, cell cycle genes exhibit significantly lower intrinsic noise but significantly higher extrinsic noise [63]. Fifth, one may already sense from the discussion of members of the same protein complex that they ideally should have not only low intrinsic noise but also coordinated expression fluctuations to attain dose balance. We found that genes located on the same chromosome tend to co-fluctuate in expression when compared with unlinked genes [64]. Interestingly, genes encoding components of the same protein complex tend to be chromosomally linked, likely resulting from natural selection for intracellular among-component dose balance [64]. More strikingly, functionally related genes (*e.g.*, those encoding enzymes in the same metabolic pathway) tend to be chromosomally clustered in eukaryotic genomes even after the exclusion of tandem duplicates [103]. Because the stochastic expression fluctuations of neighboring genes may be synchronized by shared chromatin dynamics, protein products are presumably better dose balanced when the genes are adjacent than when they are far apart on the same chromosome. We hypothesized that this could be the reason why functionally related genes tend to be neighbors on a chromosome [104]. Indeed, our manipulative experiments on three chromosomally adjacent genes encoding enzymes catalyzing consecutive reactions in yeast galactose catabolism unequivocally support this hypothesis [104]. Intriguingly, in this case, disorder in one biological phenomenon—gene expression noise—prompted the emergence of order in another—genome organization, by selection.

### Mutation

Because mutation and selection are commonly considered separate evolutionary forces, it may seem odd to discuss mutation in a section on selection. However, as a phenotypic trait, mutation rate is influenced by both the genotype [105] and the environment [106] so is potentially subject to natural selection. Genome sequencing has drastically improved our knowledge about the mutation rate as well as the molecular spectrum of mutation (*i.e.*, the relative frequencies of mutations among the four nucleotides). Three selections can act on mutation rate through promoting the fixations of mutation rate modifiers, which are mutations that affect the mutation rate (*e.g.*, mutations in genes controlling DNA repair). First, because of deleterious mutations, selection promotes the fixation of mutation rate modifiers that lower the mutation rate. Note that this is a second-order selection, because the modifiers do not directly affect the fitness of the organisms carrying the modifiers but affect the number of mutations in and hence the fitness of their offspring. Second, because of advantageous mutations, selection promotes the fixation of modifiers that increase the mutation rate. This is again a second-order selection, because the modifiers only affect the number of mutations in and hence the fitness of their offspring. Third, reducing the mutation rate may be associated with a fitness cost arising from the energy and time spent on proofreading, repair, and related biological processes. In other words, there may be a cost of fidelity that creates a first-order selection for modifiers that increase the mutation rate. Furthermore, like any trait, mutation rate is also subject to mutation bias and genetic drift. It is likely that the observed mutation rate reflects a balance among mutation bias, drift, and the three selections mentioned above. The availability of mutation rate estimates for many species allows evaluating the relative importance of these forces. For instance, the drift-barrier hypothesis proposes that the mutation rate is determined by mutation bias, drift, and the second-order selection for lower mutation rates, but ignores the first-order and second-order selections for higher mutation rates [107]. By contrast, my group found in yeast that the mutation rate is subject to stabilizing selection—both increasing and decreasing the mutation rate from the observed value are selectively disfavored [108], suggesting that selections for higher mutation rates are non-negligible. Additionally, across prokaryotes and eukaryotes, mutation rates are often orders of magnitude higher than those predicted by the drift-barrier hypothesis [108].

It is commonly assumed that the molecular spectrum of mutation simply reflects biochemical properties of DNA and is not influenced by selection. We generated yeast mutants that exhibit substantial variations in mutation spectrum, suggesting that mutation spectrum is genetically determined [108]. For example, there are more mutations from G/C to A/T than from A/T to G/C in all species examined [109], yet this is not the case in one of our mutants, in which the opposite is true [108]. This finding suggests that the universal AT mutation bias is likely a result of selection. The selective agent of the mutation spectrum, however, remains unclear.

A number of authors have reported mutation rate variation among genes in the same genome. For example, my group found that genes with higher expression levels tend to have higher mutation rates presumably due to transcription-associated mutagenesis, because the R-loop formed by the binding of the nascent RNA with its DNA template exposes the non-template DNA strand to mutagens and primes unscheduled error-prone DNA synthesis [110], [111], [112]. We further found that strong folding of nascent RNA can weaken R-loops and hence decrease transcription-associated mutagenesis [113]. Are such variations in mutation rate across genes results of natural selection optimizing gene-specific mutation rates? Specifically, it has been proposed that genes that are functionally more important or constrained have lower mutation rates as a result of stronger second-order selection against mutagenesis [114], [115], [116]. However, none of such claims have stood scrutiny [112], [117], [118]. Even if functionally more important/constrained genes have lower mutation rates, this trend would be more likely a byproduct of some other processes [119] than selective optimization of gene-specific mutation rates, because such selections would be generally too weak to have an effect [112], [117], [118]. In summary, there is good evidence for selection shaping the genomic mutation rate but no unambiguous evidence for selection shaping gene-specific mutation rates.

## Outlook

Where in evolutionary biology do I expect genomics to make the biggest impact in the next decade or two? I will name three areas. First, genome sequencing of all species on Earth will drastically improve our knowledge about the living world. For example, the Earth BioGenome Project, which started in 2018, aims to sequence the genomes of all 1.8 million known eukaryotic species in 10 years [120], [121]. Although many more prokaryotic genomes (> 150,000) than eukaryotic genomes (∼ 6000) have been sequenced to date, a recent estimate showed that nearly 98% of prokaryotic taxa have yet to be sequenced [122]. Sequencing genomes of every species will provide unprecedented information allowing systematically analyzing relationships between genomic features and other features of life, generating numerous novel hypotheses for further testing. Second, genomic tools allow high-throughput measurements of many traits such as gene and protein expression levels, post-transcriptional modifications, post-translational modifications, cell morphologies, cell physiologies, and fitness. Such information from natural variants and systematically constructed mutants will help map the genotype–phenotype–fitness landscape, test the relative roles of chance and necessity in the variations and evolution of various traits, and understand and predict evolution. Last but not least, experimental evolution coupled with genomics will be a particularly powerful approach to evolutionary mechanisms. Experimental evolution uses laboratory or controlled field manipulations to investigate evolutionary processes [123]. Because of their short generation times and small body sizes, microbes are the favored subjects of experimental evolution, although animals and plants have also been used occasionally in experimental evolution [123]. It is not an exaggeration that experimental evolution elevates evolutionary biology from an observational science to an experimental science that enables directly testing causality in evolutionary processes. Genome sequencing of strains derived from experimental evolution has already offered new insights into some evolutionary processes [79], [124], [125], [126], but genomic tools can do more than genome sequencing (*e.g.*, phenotyping molecular traits). Well-designed experimental evolution, coupled with genotyping and phenotyping of ancestral and evolved strains, will likely play an even more important role in the future development of evolutionary biology.

## CRediT authorship contribution statement

**Jianzhi Zhang:** Writing – original draft, Writing – review & editing. The author has read and approved the final manuscript.

## Competing interests

The author declares no conflict of interest.

## References

[1] Fleischmann R.D., Adams M.D., White O., Clayton R.A., Kirkness E.F., Kerlavage A.R. (1995). Whole-genome random sequencing and assembly of *Haemophilus influenzae* Rd. Science.

[2] Zhang J., Nei M. (1996). Evolution of Antennapedia-class homeobox genes. Genetics.

[3] Martin K.J., Holland P.W. (2014). Enigmatic orthology relationships between Hox clusters of the African butterfly fish and other teleosts following ancient whole-genome duplication. Mol Biol Evol.

[4] Hillis D.M., Moritz C., Mable B.K. (1996).

[5] Cheon S., Zhang J., Park C. (2020). Is phylotranscriptomics as reliable as phylogenomics?. Mol Biol Evol.

[6] Delsuc F., Brinkmann H., Philippe H. (2005). Phylogenomics and the reconstruction of the tree of life. Nat Rev Genet.

[7] Jarvis E.D. (2016). Perspectives from the avian phylogenomics project: questions that can be answered with sequencing all genomes of a vertebrate class. Annu Rev Anim Biosci.

[8] Murphy W.J., Foley N.M., Bredemeyer K.R., Gatesy J., Springer M.S. (2021). Phylogenomics and the genetic architecture of the placental mammal radiation. Annu Rev Anim Biosci.

[9] Qian W., Yang J.R., Pearson N.M., Maclean C., Zhang J. (2012). Balanced codon usage optimizes eukaryotic translational efficiency. PLoS Genet.

[10] Weinberg D.E., Shah P., Eichhorn S.W., Hussmann J.A., Plotkin J.B., Bartel D.P. (2016). Improved ribosome-footprint and mRNA measurements provide insights into dynamics and regulation of yeast translation. Cell Rep.

[11] Mordret E., Dahan O., Asraf O., Rak R., Yehonadav A., Barnabas G.D. (2019). Systematic detection of amino acid substitutions in proteomes reveals mechanistic basis of ribosome rrrors and selection for translation fidelity. Mol Cell.

[12] Sun M., Zhang J. (2022). Preferred synonymous codons are translated more accurately: proteomic evidence, among-species variation, and mechanistic basis. Sci Adv.

[13] Seehausen O., Butlin R.K., Keller I., Wagner C.E., Boughman J.W., Hohenlohe P.A. (2014). Genomics and the origin of species. Nat Rev Genet.

[14] Wu C.I., Wang H.Y., Ling S., Lu X. (2016). The ecology and evolution of cancer: the ultra-microevolutionary process. Annu Rev Genet.

[15] Bhattacharyya M.K., Smith A.M., Ellis T.H., Hedley C., Martin C. (1990). The wrinkled-seed character of pea described by Mendel is caused by a transposon-like insertion in a gene encoding starch-branching enzyme. Cell.

[16] Fishman L., McIntosh M. (2019). Standard deviations: the biological bases of transmission ratio distortion. Annu Rev Genet.

[17] Bennett G.M., Moran N.A. (2013). Small, smaller, smallest: the origins and evolution of ancient dual symbioses in a phloem-feeding insect. Genome Biol Evol.

[18] Han K., Li Z.F., Peng R., Zhu L.P., Zhou T., Wang L.G. (2013). Extraordinary expansion of a *Sorangium cellulosum* genome from an alkaline milieu. Sci Rep.

[19] Hidalgo O., Pellicer J., Christenhusz M., Schneider H., Leitch A.R., Leitch I.J. (2017). Is there an upper limit to genome size?. Trends Plant Sci.

[20] McCutcheon J.P., Moran N.A. (2011). Extreme genome reduction in symbiotic bacteria. Nat Rev Microbiol.

[21] Lynch M. (2007).

[22] Piovesan A., Antonaros F., Vitale L., Strippoli P., Pelleri M.C., Caracausi M. (2019). Human protein-coding genes and gene feature statistics in 2019. BMC Res Notes.

[23] Adams M.D., Celniker S.E., Holt R.A., Evans C.A., Gocayne J.D., Amanatides P.G. (2000). The genome sequence of *Drosophila melanogaster*. Science.

[24] FANTOM Consortium and the RIKEN PMI and CLST (DGT); Forrest AR, Kawaji H, Rehli M, Baillie JK, de Hoon MJ, et al. A promoter-level mammalian expression atlas. Nature 2014;507:462–70. 10.1038/nature13182 PMC4529748 24670764

[25] Derti A., Garrett-Engele P., Macisaac K.D., Stevens R.C., Sriram S., Chen R. (2012). A quantitative atlas of polyadenylation in five mammals. Genome Res.

[26] Ji P., Wu W., Chen S., Zheng Y., Zhou L., Zhang J. (2019). Expanded expression landscape and prioritization of circular RNAs in mammals. Cell Rep.

[27] Benne R., Van den Burg J., Brakenhoff J.P., Sloof P., Van Boom J.H., Tromp M.C. (1986). Major transcript of the frameshifted *coxII* gene from trypanosome mitochondria contains four nucleotides that are not encoded in the DNA. Cell.

[28] Christofi T., Zaravinos A. (2019). RNA editing in the forefront of epitranscriptomics and human health. J Transl Med.

[29] Boccaletto P., Stefaniak F., Ray A., Cappannini A., Mukherjee S., Purta E. (2022;50). MODOMICS: a database of RNA modification pathways. 2021 update. Nucleic Acids Res.

[30] Li S., Mason C.E. (2014). The pivotal regulatory landscape of RNA modifications. Annu Rev Genomics Hum Genet.

[31] Xu G., Zhang J. (2015). In search of beneficial coding RNA editing. Mol Biol Evol.

[32] Bazak L., Haviv A., Barak M., Jacob-Hirsch J., Deng P., Zhang R. (2014). A-to-I RNA editing occurs at over a hundred million genomic sites, located in a majority of human genes. Genome Res.

[33] Kearse M.G., Wilusz J.E. (2017). Non-AUG translation: a new start for protein synthesis in eukaryotes. Genes Dev.

[34] Lee S., Liu B., Lee S., Huang S.X., Shen B., Qian S.B. (2012). Global mapping of translation initiation sites in mammalian cells at single-nucleotide resolution. Proc Natl Acad Sci U S A.

[35] Dunn J.G., Foo C.K., Belletier N.G., Gavis E.R., Weissman J.S. (2013). Ribosome profiling reveals pervasive and regulated stop codon readthrough in *Drosophila melanogaster*. Elife.

[36] Zhang J., Xu C. (2022). Gene product diversity: adaptive or not?. Trends Genet.

[37] Meer K.M., Nelson P.G., Xiong K., Masel J. (2020). High transcriptional error rates vary as a function of gene expression level. Genome Biol Evol.

[38] Kimura M. (1983).

[39] Hurst L.D., Smith N.G. (1999). Do essential genes evolve slowly?. Curr Biol.

[40] Wang Z., Zhang J. (2009). Why is the correlation between gene importance and gene evolutionary rate so weak?. PLoS Genet.

[41] Pal C., Papp B., Hurst L.D. (2001). Highly expressed genes in yeast evolve slowly. Genetics.

[42] Zhang J., Yang J.R. (2015). Determinants of the rate of protein sequence evolution. Nat Rev Genet.

[43] Drummond D.A., Wilke C.O. (2008). Mistranslation-induced protein misfolding as a dominant constraint on coding-sequence evolution. Cell.

[44] Yang J.R., Zhuang S.M., Zhang J. (2010). Impact of translational error-induced and error-free misfolding on the rate of protein evolution. Mol Syst Biol.

[45] Yang J.R., Liao B.Y., Zhuang S.M., Zhang J. (2012). Protein misinteraction avoidance causes highly expressed proteins to evolve slowly. Proc Natl Acad Sci U S A.

[46] Shen X., Song S., Li C., Zhang J. (2022). Synonymous mutations in representative yeast genes are mostly strongly non-neutral. Nature.

[47] Li C., Wang Z., Zhang J. (2013). Toward genome-wide identification of Bateson-Dobzhansky-Muller incompatibilities in yeast: a simulation study. Genome Biol Evol.

[48] Kondrashov A.S. (1988). Deleterious mutations and the evolution of sexual reproduction. Nature.

[49] Braun P., Tasan M., Dreze M., Barrios-Rodiles M., Lemmens I., Yu H. (2009). An experimentally derived confidence score for binary protein-protein interactions. Nat Methods.

[50] Chen Y.C., Rajagopala S.V., Stellberger T., Uetz P. (2010). Exhaustive benchmarking of the yeast two-hybrid system. Nat Methods.

[51] Qian W., He X., Chan E., Xu H., Zhang J. (2011). Measuring the evolutionary rate of protein-protein interaction. Proc Natl Acad Sci U S A.

[52] Chen X., Zhang J. (2012). The ortholog conjecture is untestable by the current gene ontology but is supported by RNA sequencing data. PLoS Comput Biol.

[53] He X., Zhang J. (2005). Rapid subfunctionalization accompanied by prolonged and substantial neofunctionalization in duplicate gene evolution. Genetics.

[54] Qian W., Liao B.Y., Chang A.Y., Zhang J. (2010). Maintenance of duplicate genes and their functional redundancy by reduced expression. Trends Genet.

[55] Kuzmin E., VanderSluis B., Nguyen Ba A.N., Wang W., Koch E.N., Usaj M. (2020). Exploring whole-genome duplicate gene retention with complex genetic interaction analysis. Science.

[56] Jeong H., Mason S.P., Barabasi A.L., Oltvai Z.N. (2001). Lethality and centrality in protein networks. Nature.

[57] He X., Zhang J. (2006). Why do hubs tend to be essential in protein networks?. PLoS Genet.

[58] Wang Z., Zhang J. (2007). In search of the biological significance of modular structures in protein networks. PLoS Comput Biol.

[59] Chen P., Michel A.H., Zhang J. (2022). Transposon insertional mutagenesis of diverse yeast strains suggests coordinated gene essentiality polymorphisms. Nat Commun.

[60] Papp B., Pal C., Hurst L.D. (2003). Dosage sensitivity and the evolution of gene families in yeast. Nature.

[61] Qian W., Zhang J. (2008). Gene dosage and gene duplicability. Genetics.

[62] Lin F., Xing K., Zhang J., He X. (2012). Expression reduction in mammalian X chromosome evolution refutes Ohno’s hypothesis of dosage compensation. Proc Natl Acad Sci U S A.

[63] Sun M., Zhang J. (2020). Allele-specific single-cell RNA sequencing reveals different architectures of intrinsic and extrinsic gene expression noises. Nucleic Acids Res.

[64] Sun M., Zhang J. (2019). Chromosome-wide co-fluctuation of stochastic gene expression in mammalian cells. PLoS Genet.

[65] Li C., Qian W., Maclean M., Zhang J. (2016). The fitness landscape of a tRNA gene. Science.

[66] Puchta O., Cseke B., Czaja H., Tollervey D., Sanguinetti G., Kudla G. (2016). Network of epistatic interactions within a yeast snoRNA. Science.

[67] Bank C., Hietpas R.T., Jensen J.D., Bolon D.N. (2015). A systematic survey of an intragenic epistatic landscape. Mol Biol Evol.

[68] Olson C.A., Wu N.C., Sun R. (2014). A comprehensive biophysical description of pairwise epistasis throughout an entire protein domain. Curr Biol.

[69] Melamed D., Young D.L., Gamble C.E., Miller C.R., Fields S. (2013). Deep mutational scanning of an RRM domain of the *Saccharomyces cerevisiae* poly(A)-binding protein. RNA.

[70] Costanzo M., VanderSluis B., Koch E.N., Baryshnikova A., Pons C., Tan G. (2016). A global genetic interaction network maps a wiring diagram of cellular function. Science.

[71] He X., Qian W., Wang Z., Li Y., Zhang J. (2010). Prevalent positive epistasis in *Escherichia coli* and *Saccharomyces cerevisiae* metabolic networks. Nat Genet.

[72] Lyons D.M., Zou Z., Xu H., Zhang J. (2020). Idiosyncratic epistasis creates universals in mutational effects and evolutionary trajectories. Nat Ecol Evol.

[73] Wei X., Zhang J. (2018). The optimal mating distance resulting from heterosis and genetic incompatibility. Sci Adv.

[74] Wagner G.P., Zhang J. (2011). The pleiotropic structure of the genotype-phenotype map: the evolvability of complex organisms. Nat Rev Genet.

[75] Qian W., Ma D., Xiao C., Wang Z., Zhang J. (2012). The genomic landscape and evolutionary resolution of antagonistic pleiotropy in yeast. Cell Rep.

[76] Li C., Zhang J. (2018). Multi-environment fitness landscapes of a tRNA gene. Nat Ecol Evol.

[77] Wei X., Zhang J. (2017). The genomic architecture of interactions between natural genetic polymorphisms and environments in yeast growth. Genetics.

[78] Wei X., Zhang J. (2019). Environment-dependent pleiotropic effects of mutations on the maximum growth rate *r* and carrying capacity *K* of population growth. PLoS Biol.

[79] Chen P., Zhang J. (2020). Antagonistic pleiotropy conceals molecular adaptations in changing environments. Nat Ecol Evol.

[80] Zhang J. (2008). Positive selection, not negative selection, in the pseudogenization of *rcsA* in *Yersinia pestis*. Proc Natl Acad Sci U S A.

[81] Bakewell M.A., Shi P., Zhang J. (2007). More genes underwent positive selection in chimpanzee evolution than in human evolution. Proc Natl Acad Sci U S A.

[82] Kern A.D., Hahn M.W. (2018). The neutral theory in light of natural selection. Mol Biol Evol.

[83] Jensen J.D., Payseur B.A., Stephan W., Aquadro C.F., Lynch M., Charlesworth D. (2019). The importance of the Neutral Theory in 1968 and 50 years on: a response to Kern and Hahn 2018. Evolution.

[84] Zhang J., Kumar S. (1997). Detection of convergent and parallel evolution at the amino acid sequence level. Mol Biol Evol.

[85] Zou Z, Zhang J. The nature and phylogenomic impact of sequence convergence. In: Scornavacca C, Delsuc F, Galtier N, editors. Phylogenetics in the Genomic Era. Authors open access book 2020;4.6:1–17.

[86] Zou Z., Zhang J. (2015). Are convergent and parallel amino acid substitutions in protein evolution more prevalent than neutral expectations?. Mol Biol Evol.

[87] Li Y., Liu Z., Shi P., Zhang J. (2010). The hearing gene *Prestin* unites echolocating bats and whales. Curr Biol.

[88] Liu Y., Cotton J.A., Shen B., Han X., Rossiter S.J., Zhang S. (2010). Convergent sequence evolution between echolocating bats and dolphins. Curr Biol.

[89] Liu Z., Qi F.Y., Zhou X., Ren H.Q., Shi P. (2014). Parallel sites implicate functional convergence of the hearing gene *prestin* among echolocating mammals. Mol Biol Evol.

[90] Zhang J. (2006). Parallel adaptive origins of digestive RNases in Asian and African leaf monkeys. Nat Genet.

[91] Parker J., Tsagkogeorga G., Cotton J.A., Liu Y., Provero P., Stupka E. (2013). Genome-wide signatures of convergent evolution in echolocating mammals. Nature.

[92] Zou Z., Zhang J. (2015). No genome-wide protein sequence convergence for echolocation. Mol Biol Evol.

[93] Thomas G.W., Hahn M.W. (2015). Determining the null model for detecting adaptive convergence from genomic data: a case study using echolocating mammals. Mol Biol Evol.

[94] Zhang J. (2003). Parallel functional changes in the digestive RNases of ruminants and colobines by divergent amino acid substitutions. Mol Biol Evol.

[95] Storz J.F. (2016). Causes of molecular convergence and parallelism in protein evolution. Nat Rev Genet.

[96] He Z., Xu S., Shi S. (2020). Adaptive convergence at the genomic level—prevalent, uncommon or very rare?. Natl Sci Rev.

[97] Newman J.R., Ghaemmaghami S., Ihmels J., Breslow D.K., Noble M., DeRisi J.L. (2006). Single-cell proteomic analysis of *S. cerevisiae* reveals the architecture of biological noise. Nature.

[98] Taniguchi Y., Choi P.J., Li G.W., Chen H., Babu M., Hearn J. (2010). Quantifying *E. coli* proteome and transcriptome with single-molecule sensitivity in single cells. Science.

[99] Wang Z., Zhang J. (2011). Impact of gene expression noise on organismal fitness and the efficacy of natural selection. Proc Natl Acad Sci U S A.

[100] Lehner B. (2008). Selection to minimise noise in living systems and its implications for the evolution of gene expression. Mol Syst Biol.

[101] Chen X., Zhang J. (2016). The genomic landscape of position effects on protein expression level and noise in yeast. Cell Syst.

[102] Zhang Z., Qian W., Zhang J. (2009). Positive selection for elevated gene expression noise in yeast. Mol Syst Biol.

[103] Lee J.M., Sonnhammer E.L. (2003). Genomic gene clustering analysis of pathways in eukaryotes. Genome Res.

[104] Xu H., Liu J.J., Liu Z., Li Y., Jin Y.S., Zhang J. (2019). Synchronization of stochastic expressions drives the clustering of functionally related genes. Sci Adv.

[105] Gou L., Bloom J.S., Kruglyak L. (2019). The genetic basis of mutation rate variation in yeast. Genetics.

[106] Liu H., Zhang J. (2019). Yeast spontaneous mutation rate and spectrum vary with environment. Curr Biol.

[107] Lynch M., Ackerman M.S., Gout J.F., Long H., Sung W., Thomas W.K. (2016). Genetic drift, selection and the evolution of the mutation rate. Nat Rev Genet.

[108] Liu H., Zhang J. (2021). The rate and molecular spectrum of mutation are selectively maintained in yeast. Nat Commun.

[109] Hershberg R., Petrov D.A. (2010). Evidence that mutation is universally biased towards AT in bacteria. PLoS Genet.

[110] Park C., Qian W., Zhang J. (2012). Genomic evidence for elevated mutation rates in highly expressed genes. EMBO Rep.

[111] Chen X., Zhang J. (2014). Yeast mutation accumulation experiment supports elevated mutation rates at highly transcribed sites. Proc Natl Acad Sci U S A.

[112] Chen X., Zhang J. (2013). No gene-specific optimization of mutation rate in *Escherichia coli*. Mol Biol Evol.

[113] Chen X., Yang J.R., Zhang J. (2016). Nascent RNA folding mitigates transcription-associated mutagenesis. Genome Res.

[114] Monroe J.G., Srikant T., Carbonell-Bejerano P., Becker C., Lensink M. (2022). Mutation bias reflects natural selection in *Arabidopsis thaliana*. Nature.

[115] Xia B., Yan Y., Baron M., Wagner F., Barkley D., Chiodin M. (2020). Widespread transcriptional scanning in the testis modulates gene evolution rates. Cell.

[116] Martincorena I., Seshasayee A.S., Luscombe N.M. (2012). Evidence of non-random mutation rates suggests an evolutionary risk management strategy. Nature.

[117] Liu H., Zhang J. (2022). Is the mutation rate lower in genomic regions of stronger selective constraints?. Mol Biol Evol.

[118] Liu H., Zhang J. (2020). Higher germline mutagenesis of genes with stronger testis expressions refutes the transcriptional scanning hypothesis. Mol Biol Evol.

[119] Zhang J. (2022). Important genomic regions mutate less often than do other regions. Nature.

[120] Lewin H.A., Robinson G.E., Kress W.J., Baker W.J., Coddington J., Crandall K.A. (2018). Earth BioGenome Project: sequencing life for the future of life. Proc Natl Acad Sci USA.

[121] Lewin H.A., Richards S., Lieberman Aiden E., Allende M.L., Archibald J.M., Balint M. (2022). The Earth BioGenome Project 2020: starting the clock. Proc Natl Acad Sci U S A.

[122] Zhang Z., Wang J., Wang J., Wang J., Li Y. (2020). Estimate of the sequenced proportion of the global prokaryotic genome. Microbiome.

[123] Garland J.T., Rose M. (2009).

[124] Tenaillon O., Barrick J.E., Ribeck N., Deatherage D.E., Blanchard J.L., Dasgupta A. (2016). Tempo and mode of genome evolution in a 50,000-generation experiment. Nature.

[125] Good B.H., McDonald M.J., Barrick J.E., Lenski R.E., Desai M.M. (2017). The dynamics of molecular evolution over 60,000 generations. Nature.

[126] Barrick J.E., Lenski R.E. (2013). Genome dynamics during experimental evolution. Nat Rev Genet.

